# Gastrointestinal bleeding in chronic kidney disease patients: a systematic review and meta-analysis

**DOI:** 10.1080/0886022X.2023.2276908

**Published:** 2023-11-13

**Authors:** Yanshan Lin, Chunqun Li, David Waters, Chun Shing Kwok

**Affiliations:** a Department of Nephrology, The First Affiliated Hospital of Jinan University, Guangzhou, China; b Faculty of Health, Education and Life Sciences, Birmingham City University, Birmingham, UK; c Department of Cardiology, University Hospital of North Midlands NHS Trust, Stoke-on-Trent, UK

**Keywords:** Chronic kidney disease, gastrointestinal bleeding, mortality, risk factors

## Abstract

Gastrointestinal bleeding (GIB) is a major cause of mortality in patients with renal failure. We conducted a systematic review of the literature to evaluate the rates, predictors, and outcomes of GIB in patients with chronic kidney disease (CKD). A search of MEDLINE and EMBASE databases was performed, and data were extracted from relevant studies. Statistical pooling was performed to determine the rate of GIB in patients with CKD, and a random-effect meta-analysis was performed to determine the predictors of GIB and mortality in patients with GIB. Twenty-two studies were included in this review, with 7,810,273 patients with CKD included in the analysis. The pooled results of five studies suggested that the rate of GIB in patients with CKD was 2.2%, and among the studies in which patients with CKD underwent endoscopy, the pooled results for GIB were 35.8%. Receipt of dialysis (OR 14.48, 95%CI 4.96–42.32), older age (OR 1.03, 95%CI 1.02–1.05), diabetes mellitus (OR 1.30, 95%CI 1.22–1.39), history of ulcers (OR 1.53, 95%CI 1.03–2.26), and cirrhosis (OR 1.73, 95%CI 1.41–2.12) were significantly associated with GIB. The pooled results suggest a twofold increase in the odds of mortality with GIB, with significant heterogeneity (OR 2.12, 95%CI 1.45–3.10, *I*^2^ = 93%). GIB in patients with CKD affects 2% of patients but can be greater in the group of patients who underwent endoscopy. Receipt of dialysis is a strong predictor of GIB, and sustained GIB is associated with a twofold increase in the odds of mortality compared to patients without GIB.

## Abstract (plain language summary)

The rate of gastrointestinal bleeding (GIB) in patients with chronic kidney disease (CKD) was 2.2%.

The rate of GIB in patients with CKD is higher in those who undergo endoscopy.

Dialysis, older age, diabetes mellitus, history of ulcers, and cirrhosis were significantly associated with GIB in CKD.

GIB in patients with CKD was associated with a twofold increase in the odds of mortality.

## Introduction

Chronic kidney disease (CKD) is defined as an abnormality in the structure or function of the kidneys affecting health for greater than three months and CKD affects approximately 9.1% of the global population [1]. The impairment of kidney function in CKD disrupts multiple physiological processes and adversely affects multiple organs [2]. As renal function declines, nitrogen metabolic wastes increase in circulation and cannot be excreted, and the uremic environment can compromise the intestinal barrier [3]. It has been suggested that patients with CKD may be at greater risk of gastric mucosa damage compared to patients with normal renal function because of systemic and local chronic circulatory failure in CKD [4].

Gastrointestinal bleeding (GIB) is a common and often underestimated medical condition that subsequently leads to increased morbidity and mortality among patients with CKD [5]. Several studies have reported the growing problem of GIB in patients with CKD [6–8]. GIB is one of the most reported causes of mortality in CKD and end-stage renal disease (ESRD), accounting for 3–7% mortality of ESRD patients [9–11]. Moreover, long-term hemodialysis treatment is associated with much higher rates of mortality and morbidity compared to the general population, and studies have reported that patients receiving hemodialysis have a higher risk of bleeding [12,13]. The true morbidity and mortality associated with GIB in patients with CKD is still unclear [14].

The cause of bleeding in CKD patients remains unknown. Several studies have examined the risk factors for GIB in patients undergoing hemodialysis, with gastric ulcers being the main cause of bleeding [15]. In addition, the use of anticoagulants, platelet dysfunction, and anticoagulation during dialysis have been proposed to be contributing factors [16,17]. Among hemodialysis patients, there is conflicting evidence as one study suggests that patients on hemodialysis are more likely to have gastric ulcers than non-dialysis patients [12], while in another study, the authors found that patients on hemodialysis do not appear to be more likely to have gastric ulcers than the rest of the population [18].

The global burden of CKD is growing, and there is growing interest in the impact of GIB in this population. To date, no systematic review has evaluated the incidence of GIB in patients with CKD, factors associated with GIB in patients with CKD, and outcomes associated with patients with GIB who have underlying CKD. Therefore, we conducted a systematic review and meta-analysis of the event rates, predictors, and outcomes of patients with and without GIB in the CKD population.

## Methods

This systematic review and meta-analysis was conducted and reported according to the recommendation of the MOOSE statement [19]. The systematic review was registered in PROSPERO (CRD42023402889).

### Literature search

We used the OVID platform to search for relevant studies in MEDLINE and EMBASE in September 2023. The search terms used were: ‘(gastrointestinal bleed* or gastrointestinal hemorrhage or gastrointestinal haemorrhage) AND (chronic kidney disease or chronic renal disease or chronic renal impairment or chronic renal insufficiency or CKD).’

### Study inclusion and exclusion criteria

We included studies that evaluated GIB in patients with CKD. There was no restriction based on how GIB was identified among patients in the studies, which could be from discharge summary codes, medical records, or on endoscopic evaluation of the gastrointestinal tract. Also, there was no restriction on the definition for CKD that included patients with the diagnosis as well as patients who receive dialysis. This approach was used so that we could gather the evidence from all studies related to the topic and capture methodological heterogeneity among the existing literature. Only adult patients were included, defined as individuals aged 18 years or older. There were no restrictions on language publications. Studies with no original data, such as letters, editorials, comments, case reports, protocols, and reviews, were excluded. The reference lists of relevant studies with no actual data will be reviewed for additional studies.

### Study selection, data extraction, and quality assessment

The search results were screened to identify studies that met the inclusion criteria. The relevant studies were retrieved and downloaded.

Screening, data extraction, and quality assessment were performed independently by two reviewers (YSL and CQL). When discrepancies in study selection or data extraction were found, consensus was reached by considering the opinion of a third reviewer (CSK). Data were extracted on the study design, population, event rates of GIB, factors associated with GIB, and outcomes associated with GIB.

We used the ROBINS-I tool for the quality assessment of included studies [20]. The areas assessed included those in the preintervention stage, at intervention and post intervention and classified the overall risk of bias. The bias due to confounding and bias in selection of participants was based on whether there were differences in characteristics between patients with and GIB and whether the cohort was a non-selected CKD cohort or not. The focus of the work was on GIB rather than a typical intervention. The other post interventional potential sources of bias were deviations from intended intervention, bias due to missing data, bias in measurement of outcome, and selection of reported results.

### Data synthesis

Statistical pooling using the methods of Kwok et al. [21] was used to determine the rate of GIB events in the group with CKD and the group with angiodysplasia-related GIB. Event rates were determined in the pooled analysis, which was defined by number of GIB events in patients with CKD divided by the total number of patients with CKD. The other outcome was mortality, which was defined by death at any follow-up time point divided by the total number of patients. This mortality rate was determined for patients with CKD with or without GIB. Further pooling was performed to evaluate gastrointestinal ulcers and angiodysplasia in the overall cohort and the cohorts, excluding patients with uremia. RevMan 5.4 (Nordic Cochrane Group, Copenhagen, The Cochrane Collaboration, Copenhagen, Denmark) was used for the random-effects model with the inverse variance method to evaluate the predictors of GIB and the odds of mortality with GIB. Statistical heterogeneity was evaluated using the *I*^2^ statistic, and values of *I*^2^ of 30–60% were considered to represent a moderate degree of statistical heterogeneity [22]. In cases where there was significant heterogeneity, sensitivity analysis was performed to explore the potential sources of statistical heterogeneity with leave-one-out analysis and subgroup analysis based on factors such as patients who are receiving dialysis or those with less or more severe CKD.

## Results

After excluding those studies which did not meet the inclusion criteria, 22 studies were included in the analysis [12,13,23–42].

**Figure 1.**
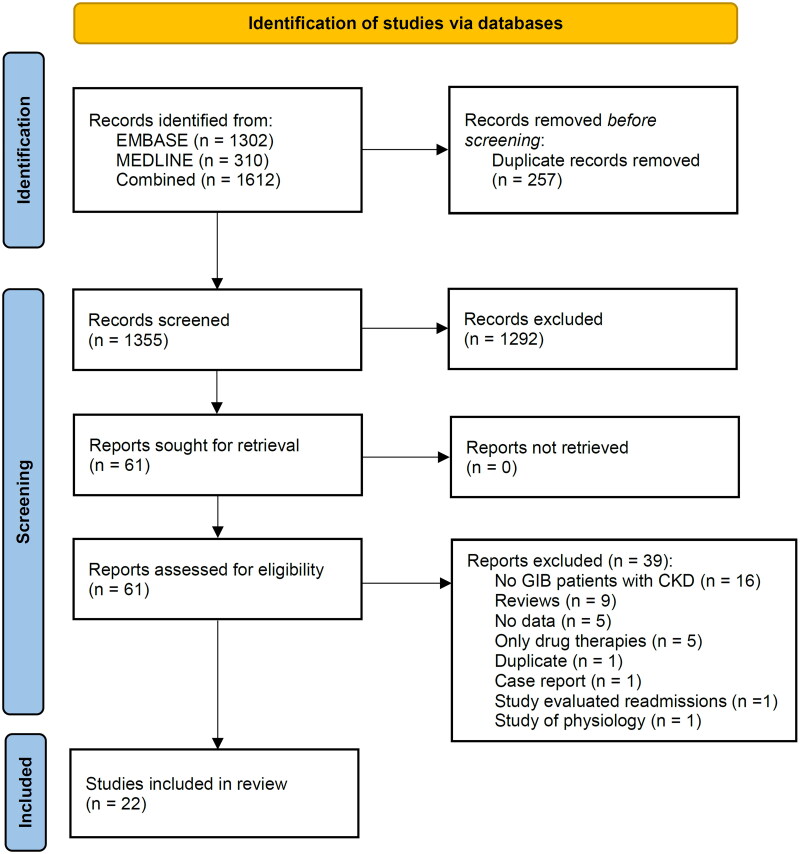
PRISMA flow diagram of study selection.

The detailed selection process is illustrated in Figure 1. Descriptions of the study design, patients, and participant inclusion criteria for the included studies are shown in Table 1. There were two prospective cohort studies, 17 retrospective cohort studies, two cross-sectional studies, and one post hoc analysis of a randomized controlled trial. There were 7,810,273 patients with CKD included in the analysis, and 11 of the studies included of patients who underwent endoscopy and these studies included 1732 patients. The average age was 61.7 years for the 15 studies that reported the mean age and the average proportion of male patients was 56.5% in 18 studies, which reported the proportion of male patients.

**Table 1.** Study design and participant inclusion criteria for studies that evaluated gastrointestinal bleeding in patients with chronic kidney disease.

Study ID	Study design; year; country	No. of participants	Mean age	% male	Participant inclusion criteria
Ali et al. [23]	Retrospective cohort study; 2007; United States.	1,799,785	–	–	Patients aged 18 years or more with discharge diagnosis of CKD from ICD-9 codes in the National Inpatient Sample.
Bang et al. [24]	Retrospective cohort study; 2003–2010; South Korea.	72 with CKD and peptic ulcer GIB	63.9	72.2%	Patients with CKD who underwent endoscopic therapy for non-variceal upper GIB.
Chacaltana et al. [25]	Retrospective cohort study; 2000–2007; Peru.	54 with end-stage renal disease	73.2	59.3%	Patients aged 18 years and over with end-stage renal disease who underwent endoscopy.
Daud et al. [26]	Cross-sectional study; 2016; Malaysia.	171 with CKD and anemia	All patients >60 years	56.7%	Patients aged 60 years and over with CKD stage 3–5 with anemia who underwent endoscopy.
Delsa et al. [27]	Cross-sectional study; 2007–2013; Morocco.	372	44.7	54.8%	Patients with CKD and upper gastrointestinal endoscopy.
Docherty et al. [28]	Retrospective cohort study; 2005–2012; United Kingdom.	69	–	–	Patients with small bowel capsule endoscopy who had estimated glomerular filtration rate <60 ml/min/1.73 m^2^.
Hanouneh et al. [29]	Retrospective cohort study; 2002–2013; United States.	66	–	–	Adult patients with CKD stage 3–5 with obscure GIB who underwent double-balloon enteroscopy.
Hung et al. [30]	Retrospective cohort study; 2010–2017; Taiwan.	78 uremic patients with obscure GIB	69.2	26.9%	Patients with obscure GIB who underwent single-balloon enteroscopy.
Jamal et al. [31]	Retrospective cohort study; 2011–2014; Morocco.	183	57.6	78.7%	Patients with chronic renal insufficiency presenting with GIB who underwent high digestive endoscopy.
Kim et al. [32]	Retrospective cohort study; 2003–2016; South Korea.	230	65.7	47.9%	Patients with CKD who underwent colonoscopy due to suspected lower GIB.
Liang et al. [33]	Prospective cohort study; 2003–2012; Taiwan.	2968	65.5	54.4%	Patients with stage 3–5 CKD in an outpatient-based CKD program.
Liang et al. [34]	Prospective cohort study; 2003–2012; Taiwan.	3126 patients with CKD and not on dialysis	65	54.1%	Patients with stage 3–5 CKD and not on dialysis in an outpatient-based CKD program.
Little et al. [35]	Retrospective cohort study; 2004–2017; United Kingdom.	310,953	Median 76	60.4%	Patients aged 18 years or more with CKD in the UK Clinical Practice Research Datalink.
Luo et al. [13]	Retrospective cohort study; 2000–2006; Taiwan.	36,474 with dialysis, 6320 with CKD, 36,034 control.	63.0	49.1%	Patients were in the National Health Insurance Research Database in Taiwan with end-stage renal disease on hemodialysis, CKD, and matched controls.
Luo et al. [12]	Retrospective cohort study; 2000–2006; Taiwan.	8210 with hemodialysis, 4190 with CKD, 8430 control.	66.2 (CKD and hemodialysis patients)	56.9% (CKD and hemodialysis patients)	Patients were in the National Health Insurance Research Database in Taiwan with end-stage renal disease on hemodialysis, CKD, and matched controls.
Mahady et al. [36]	Post hoc analysis of randomized controlled trial; 2010–2017; United States.	4592 with CKD and 13,024 control.	75	44%	Patients were aged 70 years and older in the ASPREE clinical trial.
Mandava et al. [37]	Retrospective cohort study; 2008–2012; United States.	327	62.0	55.4%	Patients with CKD stage 3–5 and above with esophagogastroduodenoscopy.
Oliveira et al. [38]	Retrospective cohort study; 1990; Portugal.	301	57.4	55.1%	Patients with CKD and dialysis.
Prasad et al. [39]	Retrospective cohort study; 2011–2012; India.	110	35.0	81.8%	Patients with CKD who attended hospital and were on the endoscopic registers and case study books of CKD.
Randhawa et al. [40]	Retrospective cohort study; 2014; United States.	124,648 with CKD (4787 GIB).	–	–	Adult patients in the Healthcare Cost and Utilization Project’s National Inpatient Sample with CKD and GIB.
Tariq et al. [41]	Retrospective cohort study; 2009–2014; United States.	5,505,252 CKD (24,709 angiodysplasia GIB).	22.1 > 75 years	52.8%	Adult patients in the National Inpatient Sample with angiodysplasia associated GIB and end-stage renal disease.
Tsai et al. [42]	Retrospective cohort study; 2000–2012; Taiwan.	574 dialysis, 1148 CKD, 1148 control.	61.7 (dialysis and CKD)	56.4% (dialysis and CKD)	Patients were age 20 years or older with CKD with and without dialysis in the National Health Insurance Database on Taiwan.

CKD: chronic kidney disease; GIB: gastrointestinal bleeding; ICD: International Classification of Disease.

### Quality assessment of included studies

The risk of bias assessment for the included studies using the ROBIN-I tool is shown in Supplementary Table 1. A total of 13 studies were classified as having low risk of bias while nine studies were classified as having moderate or serious risk of bias.

### Results of gastrointestinal bleeding events and findings from the subgroup that underwent endoscopy

The follow-up results of the included studies are shown in Table 2. The pooled results of five studies suggested that the rate of GIB in patients with CKD was 2.2% (43,702/1,970,654), and the pooled results for two studies of the rate of angiodysplasia GIB were 0.4% (24,746/5,506,974). The study by Little et al. reported the rate of GIB in patients with dialysis dependent CKD as 5.74 per 100 person years and in non-dialysis dependent CKD as 2.42 per 100 person years [35]. In the study by Mahady et al., GIB occurred in 4.6 vs. 2.5 per 1000 person years for patients with CKD vs. no CKD, respectively and there was increased risk of GIB with CKD (RR 1.90, 95%CI 1.40–2.40, *p* < .001) [36]. Among the studies in which patients with CKD underwent endoscopy, the pooled results for GIB were 35.8% (272/760, seven studies), and the pooled results for gastrointestinal ulcer and angiodysplasia were 14.2% (155/1093, seven studies) and 14.3% (138/968, six studies), respectively. Excluding a single study of uremic patients with CKD, the rates of GIB, gastrointestinal ulcer, and angiodysplasia were a lower rate of 31.2%, 14.9%, and 10.6%, respectively.

**Table 2.** Studies that evaluated gastrointestinal bleeding events in patients with chronic kidney disease.

Study ID	Follow up	Results
Ali et al. [23]	In-hospital events.	GIB events: 35,985/1,799,785 hospital patients with CKD.
Chacaltana et al. [25]	In-hospital events.	GIB events: 16/54. Endoscopic lesions: gastric erosion 19/54, gastric ulcer 13/54, duodenal erosion 10/54, duodenal ulcer 4/54, gastric angiodysplasia 4/54, erosive esophagitis 3/54, and Mallory-Weiss tear 1/54.
Daud et al. [26]	None (cross-sectional study)	GIB events: 85/171 with positive upper endoscopy for GIB, 22/171 with positive colonoscopy for GIB.
Delsa et al. [27]	None (cross-sectional study)	Upper GI endoscopy identified 313 lesions: 156 congestive gastritis, 58 ulcerative gastritis, 43 angiodysplasia, 26 peptic esophagitis, 20 signs of portal hypertension, 18 peptic ulcer, and eight hiatus hernia.
Docherty et al. [28]	In-hospital events.	Capsule endoscopy 17/51 angioectasia, 2/51 bleeding, and 1/51 adenocarcinoma.
Hanouneh et al. [29]	In-hospital events.	Arteriovenous malformation 30/66. Bleeding due to erosions 7/66 and ulcers 7/66.
Hung et al. [30]	In-hospital events.	Uremic patients with dialysis diagnostic yield of single balloon enteroscopy 59/78. Location of bleeding: stomach 4/78, duodenum 9/78, jejunum 30/78, ilium 11/78, colon 5/78. Causes of bleeding: angiodysplasia 44/78, ulcer 4/78, tumor 2/78, diverticulum 6/78, and other 3/78.
Jamal et al. [31]	In-hospital events.	Gastroscopy bulbar ulcer 81/183, erosive gastritis 57/183, peptic esophagitis 27/183, and angiodysplasia 18/183.
Kim et al. [32]	In-hospital events.	Colonoscopy 73/230 had confirmed lower GIB. Causes of bleeding: hemorrhoids (32/230), colorectal ulcer (21/230), diverticular bleeding (12/230), colitis (12/230), and angiodysplasia (12/230). Progression of CKD stage associated with increased lower GIB (*p* = .002). Lower GIB associated with hemodialysis (*p* = .001) and hypoalbuminemia (*p* = .002).
Liang et al. [33]	Median 1.9 years follow up.	Rate of upper GIB 386/2968.
Liang et al. [34]	2.8 years.	Rate of upper GIB: 387/3126.
Little et al. [35]	Follow up range for mortality was 1–5 years.	Rate of GIB per 100 person years: Dialysis-dependent CKD: 5.74 Incident dialysis-dependent CKD: 6.10 Non-dialysis dependent CKD: 2.42
Luo et al. [13]	7-year period.	Ulcer bleeding: normal 480/36,034, CKD 163/6320, and hemodialysis 2361/36,474.
Luo et al. [12]	Median follow up time of 2.14 and 2.24 for hemodialysis and CKD patients.	Nonpeptic ulcer, nonvariceal GIB: normal 98/8430, CKD 189/4190, and hemodialysis 647/8210.
Mahady et al. [36]	Median follow up 4.7 years.	GIB with CKD: 4.6 per 1000 person years GIB without CKD: 2.5 per 1000 person years Risk of GIB with CKD: RR 1.90 95%CI 1.40–2.40, *p* < .001
Oliveira et al. [38]	In-hospital events.	GIB events in patients: 19/301 (23 total bleeds 19 upper GIB and four lower GIB). Causes of upper GIB were nine peptic ulcer, seven gastritis/duodenitis, one angiodysplasia, one Mallory Weiss tear, and one unknown. Causes of lower GIB were three colon angiodysplasia, one colon cancer.
Prasad et al. [39]	In-hospital events.	Events on endoscopy register: esophagitis 10/110, gastritis 20/110, duodenitis 8/110, duodenal ulcer 5/110, gastric ulcer 2/110, and upper GIB 8/110.
Randhawa et al. [40]	In-hospital events.	Prevalence of GIB in patients with CKD: 4787/124,648. Propensity score matched cohort GIB with CKD vs. no CKD: 3.1% vs. 2.5%, *p* < .001. Lower GIB bleed with CKD vs. no CKD: 0.9% vs. 0.7%, *p* < .001.
Tariq et al. [41]	In-hospital events.	Prevalence of angiodysplasia GIB in patients with end-stage renal disease: 24,709/5,505,252.
Tsai et al. [42]	Mean 6.4 years.	Propensity matched events in different groups: GIB hospitalization: dialysis 175/574, non-dialysis CKD 126/1148, and control group 93/1148. Lower gastrointestinal bleeding: dialysis 74/574, non-dialysis CKD 41/1148, and control 32/1148. Angiodysplasia bleeding: dialysis 6/574, non-dialysis CKD 1/1148, and control 1/1148.

CKD: chronic kidney disease; GIB: gastrointestinal bleeding.

### Evaluation of predictors of gastrointestinal bleeding events

**Figure 2.**
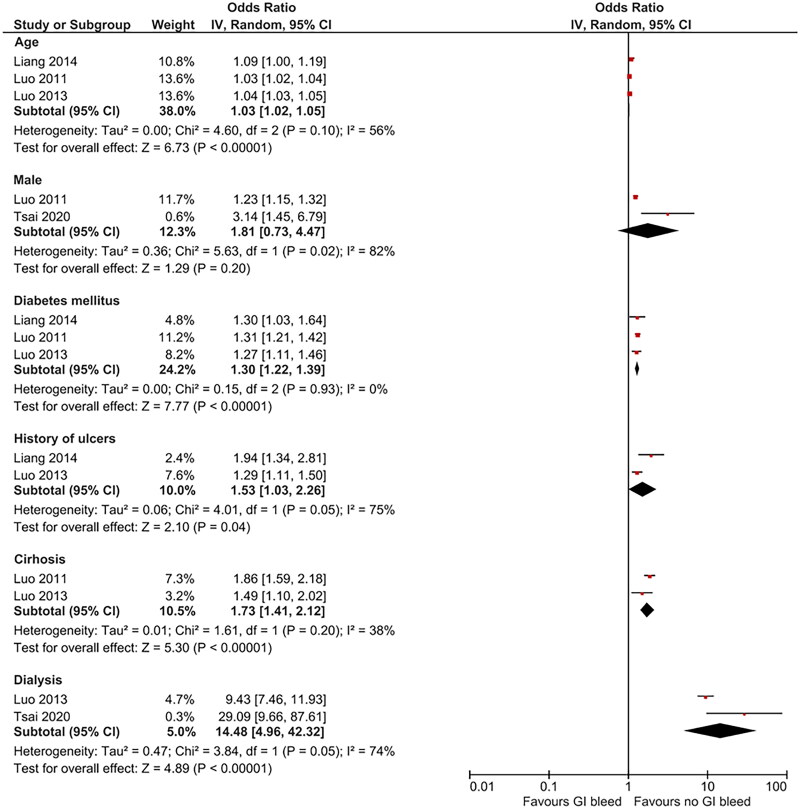
Forest plot of pooled factors associated with gastrointestinal bleeding in patients with chronic kidney disease.

The results of the studies that evaluated predictors of GIB events are shown in Table 3, and the meta-analysis of individual factors and the risk of GIB in patients with CKD are displayed in Figure 2. Receipt of dialysis (OR 14.48, 95%CI 4.96–42.32, *I*^2^ = 74%, two studies), age (OR 1.03, 95%CI 1.02–1.05, *I*^2^ = 56%, three studies), diabetes mellitus (OR 1.30, 95%CI 1.22–1.39, *I*^2^ = 0%, two studies), history of ulcers (OR 1.53, 95%CI 1.03–2.26, *I*^2^ = 75%, two studies), and cirrhosis (OR 1.73, 95%CI 1.41–2.12, *I*^2^ = 38%, two studies) were significantly associated with GIB. Other factors from single studies that were major predictors of GIB in patients with CKD undergoing hemodialysis were non-steroidal anti-inflammatory drug use (OR 1.94, 95%CI 1.76–2.13) and selective serotonin reuptake inhibitor use (OR 1.70, 95%CI 1.15–2.52).

**Table 3.** Studies that evaluated predictors of gastrointestinal bleeding events in patients with chronic kidney disease.

Study ID	Follow up	Results
Ali et al. [23]	In-hospital events.	GIB was associated with abnormal renal function aOR 1.58, 95%CI 1.43–1.76.
Bang et al. [24]	7 days for initial therapy.	Factors associated with rebleeding: alcohol OR 11.19, *p* = .02, therapy OR 0.06, *p* = .01, experience of endoscopist in years: OR 0.56, *p* = .03.
Hung et al. [30]	None	Significant risk factors for rebleeding: valvular heart disease OR 4.78, 95%CI 1.02–22.40, *p* = .047.
Liang et al. [33]	Median 1.9 years follow up.	Multivariable risk factors for upper GIB: eGFR aHR 0.90, 95%CI 0.84–0.96, age aHR 1.09, 95%CI 1.00–1.19, history of upper GIB aHR 1.94, 95%CI 1.36–2.83, diabetes mellitus aHR 1.30, 95%CI 1.03–1.63.
Luo et al. [13]	7-year period.	Predictors of ulcer bleeding: age aHR 1.03, 95%CI 1.02–1.03, male aHR 1.23, 95%CI 1.15–1.32, hypertension aHR 1.19, 95%CI 1.07–1.32, diabetes mellitus 1.31, 95%CI 1.21–1.42, coronary artery disease aHR 1.14, 95%CI 1.05–1.24, heart failure aHR 1.13, 95%CI 1.03–1.24, cirrhosis aHR 1.86, 95%CI 1.59–2.18, NSAID aHR 1.94, 95%CI 1.76–2.13, CKD aHR 1.95, 95%CI 1.62–2.35, hemodialysis aHR 5.24, 95%CI 4.67–5.86. Predictors of ulcer bleeding in dialysis patients: diabetes mellitus aHR 1.44, 95%CI 1.32–1.57, coronary artery disease aHR 1.29, 95%CI 1.18–1.41, cirrhosis aHR 1.85, 95%CI 1.57–2.18, NSAID use aHR 1.95, 95%CI 1.74–2.18.
Luo et al. [12]	7-year follow up with median follow up time of 2.14 and 2.24 for hemodialysis and CKD patients.	Predictors of nonpeptic ulcer nonvariceal GIB: age aHR 1.04, 95%CI 1.03–1.05, diabetes mellitus aHR 1.27, 95%CI 1.11–1.46, cirrhosis aHR 1.49, 95%CI 1.10–2.02, chronic obstructive pulmonary disease aHR 1.33, 95%CI 1.14–1.55, history of uncomplicated peptic ulcer disease aHR 1.29, 95%CI 1.11–1.50, CKD aHR 5.17, 95%CI 4.00–6.68, dialysis aHR 9.43, 95%CI 7.46–11.93, selective serotonin reuptake inhibitors aHR 1.70, 95%CI 1.15–2.52. Predictors of nonpeptic ulcer nonvariceal GIB in dialysis: age aHR 1.04, 95%CI 1.03–1.04, diabetes mellitus aHR 1.20, 95%CI 1.02–1.41, cirrhosis aHR 1.57, 95%CI 1.13–2.18, chronic obstructive pulmonary disease aHR 1.26, 95%CI 1.05–1.52, history of uncomplicated peptic ulcer disease aHR 1.39, 95%CI 1.17–1.64, SSRIs aHR 1.87, 95%CI 1.19–2.96.
Mandava et al. [37]	In-hospital events.	Univariable factors associated with upper GIB were cirrhosis, coumadin use, lack of proton pump inhibitor use, lower admission hemoglobin (Hb), and greater difference in baseline Hb to pre-procedure Hb (*p* < .05). The predictors of upper GIB in CKD III/IV were atrial fibrillation diagnosis and in CKD V/ESRD was HD use. The greatest predictor of an upper GIB was a greater difference in baseline Hb to pre-procedure Hb (*p* < .05).
Tariq et al. [41]	In-hospital events.	Predictors of angiodysplasia GIB: 2010 aOR 1.27, 95%CI 1.07–1.52, 2011 aOR 1.28, 95%CI 1.08–1.53, 2012 aOR 1.43, 95%CI 1.23–1.66, 2013 aOR 1.51, 95%CI 1.30–1.76, 2014 aOR 1.39, 95%CI 1.19–1.61, age vs. 18–44 reference, 45–64, 95%CI 4.12, 95%CI 3.05–5.57, 65–74, aOR 7.42, 95%CI 5.27–10.40, 75+ aOR 8.22, 95%CI 5.87–11.50, race vs. Caucasian reference, African American aOR 1.12, 95%CI 1.02–1.23, Asian Pacific Islander aOR 0.77, 95%CI 0.62–0.96, primary expected payer vs. Medicare reference, others aOR 0.69, 95%CI 0.52–0.90, Medicaid aOR 0.84, 95%CI 0.69–1.10, private payer aOR 0.96, 95%CI 0.83–1.10, self-payer aOR 0.32, 95%CI 0.20–0.51, Charlson-Deyo comorbidity index vs. 1–2 score 3–4 aOR 1.15, 95%CI 1.04–1.27, score 5+ aOR 1.26, 95%CI 1.12–1.43, hypertension aOR 2.01, 95%CI 1.79–2.26, diabetes mellitus aOR 0.79, 95%CI 0.73–0.85, tobacco use aOR 1.26, 95%CI 1.17–1.36, hospital location, teaching status vs. urban teaching reference, rural aOR 0.78, 95%CI 0.66–0.93, unknown aOR 1.20, 95%CI 0.77–1.86, urban non-teaching aOR 0.89, 95%CI 0.80–0.98. Odds of mortality with angiodysplasia GIB: OR 0.67, 95%CI 0.58–0.78.
Tsai et al. [42]	Mean 6.4 years.	Significant predictors of lower GIB: Dialysis aHR 29.09, 95%CI 9.66–87.63, non-dialysis CKD aHR 6.61, 95%CI 2.27–19.23, age >85 vs. 20–44 aHR 61.47, 95%CI 2.68–1412.10, male aHR 3.14, 95%CI 1.45–6.78.

GIB: gastrointestinal bleeding; aOR: adjusted odds ratio; aHR: adjusted hazard ratio; CKD: chronic kidney disease; SSRIs: selective serotonin reuptake inhibitors; Hb: hemoglobin.

In two studies, Bang et al. and Tariq et al. reported results that were not pooled [24,41]. Bang et al. found that alcohol (*p* = .02) and receipt of therapy (*p* = .01) were associated with increased odds of rebleeding, while the experience of the endoscopist was associated with reduced odds of bleeding [24]. Tariq et al. found that the strong factors associated with angiodysplasia GIB were old age (age 65–74 vs. 18–44 years, aOR 7.42, 95%CI 5.27–10.40, age 75+ vs. 18–44 years, aOR 8.22, 95%CI 5.87–11.50) and hypertension (aOR 2.01, 95%CI 1.79–2.26) [40].

**Figure 3.**
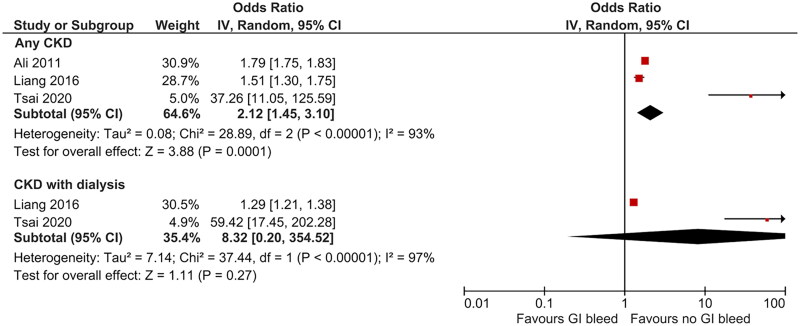
Forest plot of the meta-analysis of gastrointestinal bleeding and mortality in patients with chronic kidney disease.

Table 4 shows the results of the studies that evaluated the outcomes of patients with GIB and CKD. The pooled results of the three studies that evaluated the impact of GIB on mortality in patients with CKD are shown in Figure 3. The pooled results suggest a twofold increase in the odds of mortality with GIB, with significant heterogeneity (OR 2.12, 95%CI 1.45–3.10, *I*^2^ = 93%). While the exclusion of individual studies failed to reduce the heterogeneity significantly, all studies consistently estimated a significant increase in mortality with GIB. Among patients with CKD who received dialysis, the pooled results of two studies suggested a non-significant increase in the odds of mortality (OR, 8.32; 95%CI 0.20–354.52, *I*^2^ = 97%).

**Table 4.** Studies that reported outcomes for patients with gastrointestinal bleeding events and chronic kidney disease.

Study ID	Follow up	Results
Ali et al. [23]	In-hospital events.	In-hospital mortality for CKD with GIB vs. CKD with no GIB was 4.19% vs. 2.38%.
Bang et al. [24]	7 days for initial therapy.	Factors associated with prognosis: rebleeding OR 7.10 (*p* = .02).
Liang et al. [34]	2.8 years.	Risk of mortality with upper GIB vs. no GIB: 45/387 vs. 152/2739. Upper GIB and mortality: aHR 1.51, 95%CI 1.07–2.13. Upper GIB and mortality in dialysis patients: aHR 1.29, 95%CI 1.11–1.50.
Tariq et al. [41]	In-hospital events.	Odds of mortality with angiodysplasia GIB: OR 0.67, 95%CI 0.58–0.78.
Tsai et al. [42]	Mean 6.4 years.	Propensity matched events in different groups: Bleeding related mortality: dialysis 14/574, non-dialysis CKD 13/1148, and control 0/1148.

GIB: gastrointestinal bleeding; OR: odds ratio; aHR: adjusted hazard ratio; CKD: chronic kidney disease.

A few studies were not included in the pooled analysis of mortality due to GIB. Bang et al. found that rebleeding was a major predictor of prognosis (OR 7.10, *p* = .02). Tariq et al. reported that angiodysplasia bleeding was associated with reduced odds of mortality (OR, 0.67; 95%CI 0.58–0.78). Among patients on dialysis, Liang et al. found that GIB was associated with increased odds of mortality (OR, 1.29; 95%CI 1.11–1.50). In a study by Tsai et al., bleeding-related mortality was 2.4% for patients with dialysis and 1.1% for patients with CKD without dialysis.

## Discussion

Our study highlights the fact that GIB occurs in many patients with CKD. GIB occurs in 2% of patients with CKD in cohort studies and a much higher rate in patients who underwent endoscopic evaluation. Furthermore, many factors are associated with GIB in patients with CKD, including hemodialysis, cirrhosis, history of ulcer disease, diabetes mellitus, and older age. These findings may be important in identifying which patients may benefit from further evaluation for GIB, particularly if they also have symptoms that may be related to GIB. Another key finding is that patients with CKD and GIB have a twofold increase in the mortality risk compared to patients with CKD alone. This highlights the importance of timely identification and management of GIB in CKD patients to reduce the risk of adverse outcomes. These findings suggest that patients with CKD may be susceptible to GIB and that those who sustain GIB have worse prognosis compared to patients without GIB, and studies are needed to better understand how we can reduce these adverse events.

We identified two distinct cohorts that evaluated GIB in patients with CKD. The GIB events in patients with CKD differ considerably for the cohort of patients from hospitals and databases compared to the patients with CKD who underwent endoscopy for suspected gastrointestinal pathology, as the pooled rate of GIB was 2% compared to 35.6%. The evaluation of hospital records or registries has the advantage of capturing clinically significant GIB events that require management. However, there is no assurance that this method may capture all cases of GIB, particularly those that are minor or undiagnosed, which results in the underestimation of GIB in the CKD population. The group that underwent endoscopy, on the other hand, had more reliable ascertainment of the presence or absence of a cause for GIB. However, these patients, particularly in observational studies, must have had an indication for the endoscopy investigation, such as suspicion of GIB or pathology, as the procedure is not without risk.

The exact reason for the increased mortality in patients with GIB and CKD is unclear and likely multifactorial. Patients with CKD are frail and often have other comorbidities such as diabetes mellitus and cardiovascular diseases. In particular, patients with ischemic heart disease, stroke, and peripheral artery disease may be on antithrombotic medications, and patients who have bleeding when they have risk factors, or a history of ischemic events are difficult to manage because the risk of ischemic events needs to be weighed against the risk of bleeding. In addition, patients with CKD may have inadequate erythropoietin production, and consequential anemia and GIB may be catastrophic in patients who are already anemic. Future studies should evaluate whether any measures can be taken to reduce mortality in patients with GIB and CKD, as there are very limited studies that provide evidence as to why patients with CKD and GIB die.

The evaluation of the factors associated with GIB merits consideration. First, we found that hemodialysis was strongly associated with GIB. While the exact reason for the increased incidence of GIB in patients on long-term dialysis is not clear, it may be due to heparin use and chronic anticoagulation from hemodialysis [15] or antithrombotic medications with the management of other cardiovascular comorbid illnesses. Diabetes mellitus is also a significant predictor of GIB. Cardiovascular disease is the most frequent complication of type 2 diabetes mellitus [43] and the use of antithrombotic drugs may explain the association with GIB. A history of ulcers is not surprising to be associated with bleeding, as these lesions are predisposed to bleeding, and cirrhosis is known to be associated with esophageal variceal bleeding. Non-steroidal anti-inflammatory medications are known to cause mucosal injury in the upper, mid-, and lower gastrointestinal tract, resulting in bleeding [44], and a meta-analysis of four observational studies suggested that selective serotonin reuptake inhibitors increase the odds of upper GIB by 2.36-folds, which increase the 6.33-folds odds of concomitant non-steroidal anti-inflammatory drug use [45].

Three studies were pooled that evaluated the impact of GIB on mortality in patients with CKD and each had different follow up events from in-hospital to a mean of 6.4 years. GIB is an acute event that requires treatment and in-hospital or short-term mortality is likely related to the acute events. However, in longer-term follow-up over a year, it is possible that the outcome may only be partly related to the GIB event but more related to post-GIB events and management or the characteristics of the patients who have GIB in terms of comorbidities and frailty. Nevertheless, the direction of the effect is consistent among the studies as all suggest that GIB in patients with CKD has greater mortality and the study by Tsai et al. has much greater estimate because there is a smaller sample size and no events in the control group, which contribute to wide confidence intervals.

There are a few important considerations regarding the studies of GIB in patients with CKD. First, endoscopy is a reliable investigation to identify GIB and its etiology, but some studies evaluated discharge summaries or medical records for GIB and this is likely to miss minor bleeds and may not have information about the etiology of bleed. However, endoscopy is an invasive procedure with risks and the requirement of time, personnel, and equipment. It is not performed on every patient within the population with CKD but rather on those who have risk factors or suspected bleed. Understanding the etiology of the GIB is nevertheless important as the cause for the underlying bleed should be treated. Second, the current review appears to underreport rebleeding, highlighting the need for further investigation in future studies. Equally important for rebleeding is the identification of risk factors for bleeding so that can be targeted and managed to prevent these secondary bleeding events. Finally, the prediction of in-hospital mortality in patients with CKD and GIB is important. Awareness of risk factors for mortality may enable the identification of patients who may require measures to mitigate or prevent GIB or closer monitoring so that if bleeds occur, patients can be treated rapidly. Examples of these measures could be prophylactic proton pump inhibitor therapy or alteration in medications including the avoidance or dose reduction of non-steroid anti-inflammatory medication.

The findings of this study have some clinical implications. First, patients with CKD are at risk for GIB; therefore, measures should be taken to reduce the risk of bleeding. This may involve screening patients for risk factors for GIB and altering the management of those with risk factors. For example, there may be a role for proton pump inhibitor medication in some patients who are at risk of bleeding. Furthermore, patients at a high risk of bleeding benefit from less aggressive antithrombotic medication. Antithrombotic medications are known to predispose GIB and these agents include antiplatelet and anticoagulant therapies. For patients with multiple indications for these drugs such as atrial fibrillation or acute myocardial infarction with coronary stenting, patients may be on anticoagulation as well as dual antiplatelet therapy. Risk assessment for bleeding is important as the benefit of these medications needs to be weighed against the risk of bleeding and for some patients with CKD there may be grounds to be less aggressive with antithrombotic medication using fewer agents or less potent antiplatelet agents such as clopidogrel or aspirin rather than prasugrel or ticagrelor. In addition, exposure to certain medications that increase the propensity to bleed, such as non-steroidal anti-inflammatory and selective serotonin reuptake inhibitor medications, should be minimized.

The findings of the current study may be generalizable to patients with CKD from hospital settings and to patients with CKD who undergo endoscopy, but some of the studies were derived from specific populations. It is notable that a few studies were much larger than the other studies, and these were those derived from the National Inpatient Sample in the United States [23,38,39]. Tariq et al. also differed from the other studies as it focused on angiodysplasia GIB [41]. There were also other studies that restricted the cohort to those who underwent colonoscopy [32], capsule endoscopy [28], and two studies sampled patients from outpatient settings [33,34].

### Limitations

The systematic review and meta-analysis had several limitations. First, only a limited number of studies were included in this pooled meta-analysis. Second, the observational nature of the included studies may have been affected by measured or unmeasured confounding factors. Ten of the included studies, however, made efforts to adjust for potential confounding or used matching methods to limit the effects of confounders. Third, the majority of the studies were retrospective in design, and there may be less certainty in detecting exposure and outcomes compared to prospective studies that ascertain key variables at baseline and follow-up. However, there was a large group of studies from the endoscopy cohort that should be considered a reliable test to detect GIB. Another limitation of the current study was that we were not able to consider the impact of the antithrombotic medications, type of dialysis, duration of dialysis, and the severity of GIB and CKD. This is important as minor GIB may not be clinically significant or may be life-threatening in the case of major bleeding or have prognostic implications such as bleeding due to underlying malignancy. The severity of CKD might be important as there may be greater cardiovascular consequences for severe or end-stage CKD, which may impact risk of mortality and treatment with antithrombotic medications. Also, we did not find studies evaluating the influence of CKD on rebleeding rates and failure of endoscopic therapy. This is an area that requires further research. In the current review, we only included published literature because conference abstracts lack the detailed description to fully understand the methodology that is necessary to perform a reliable quality assessment. It is therefore a limitation that there may be possible publication bias where only studies that have positive findings are published and those with negative or unpublished findings are not captured in the review. Finally, the etiology of GIB may be relevant and of interest because it may affect management, and it was not captured or consistently reported in all studies. A subgroup of the included studies evaluated specific types of bleeds such as those related to angiodysplasia, and those that were classified as non-variceal, upper gastrointestinal, lower gastrointestinal, non-ulcer, and ulcer bleeding. The use of endoscopy in other studies was able to identify the cause of GIB, which included arteriovenous malformation, cancer, gastritis, esophagitis, and erosions.

## Conclusions

GIB affects 2% of patients with CKD, may be higher for the group of patients who have an indication to undergo endoscopy. In terms of causes of GIB, among studies with patients who undergo endoscopy, ulcer and angiodysplasia have been reported to be frequent causes. Receipt of dialysis is a strong predictor of GIB, and sustained GIB is associated with a twofold increase in the odds of mortality compared to patients without GIB. More studies are needed to determine whether GIB can be prevented in patients with CKD and what measures should be taken to reduce mortality among patients with CKD who have a GIB event.

## Supplementary Material

Supplemental Material
