# Supplementary material for: Gastrointestinal bleeding in chronic kidney disease patients: a systematic review and meta-analysis
Source: Ren Fail. 2023 Nov 13;45(2):2276908. doi: 10.1080/0886022X.2023.2276908 (PMC10796123; doi:10.1080/0886022X.2023.2276908)
Supplement: Supplemental Material [file IRNF_A_2276908_SM2372.pdf]

Supplementary Table 1: Risk of bias assessment of studies of gastrointestinal bleeding events in patients with chronic kidney disease

Study		Pre-intervention		At intervention	Post-intervention				Overall risk of bias
First author	Year	Bias due to confounding	Bias in selection of participants into the study	Bias in classification of interventions	Bias due to deviations from intended interventions	Bias due to missing data	Bias in measurement of outcomes	Bias in selection of the reported result	Low/ moderate/ serious/ critical
Ali	2011	low	low	low	low	low	low	low	low
Bang	2013	moderate	moderate	low	low	low	moderate	moderate	moderate
Chacaltana	2007	moderate	moderate	low	low	low	moderate	moderate	moderate
Daud	2022	moderate	serious	low	low	low	moderate	moderate	serious
Delsa	2013	moderate	moderate	low	low	low	moderate	moderate	moderate
Docherty	2013	moderate	serious	low	low	low	moderate	moderate	serious
Hanouneh	2017	moderate	moderate	low	low	low	moderate	moderate	moderate
Hung	2022	moderate	serious	low	low	low	low	low	serious
Jamal	2018	moderate	moderate	low	low	low	low	low	low
Kim	2017	moderate	moderate	low	low	low	low	low	low
Liang	2014	low	low	low	low	moderate	low	low	low
Liang	2016	low	low	low	low	moderate	low	low	low
Little	2023	low	low	low	low	moderate	low	low	low
Luo	2011	low	low	low	low	moderate	low	low	low
Luo	2013	low	low	low	low	low	low	low	low
Mandava	2013	low	serious	low	low	low	low	low	serious
Mahady	2023	low	moderate	low	low	low	low	low	low
Oliveira	1992	moderate	low.	low	low	low	moderate	moderate	low
Prasad	2012	moderate	moderate	low	low	low	moderate	moderate	moderate
Randhawa	2020	low	low	low	low	low	low	low	low
Tariq	2019	low	low	low	low	low	low	low	low
Tsai	2020	low	low	low	low	low	low	low	low

Information Classification: General
